# Molecular triage of cervical screening samples in women 55–59 years of age: a pilot study

**DOI:** 10.1186/s13027-023-00510-1

**Published:** 2023-05-23

**Authors:** Gisela Helenius, Gabriella Lillsunde-Larsson, Lovisa Bergengren

**Affiliations:** 1 School of Health Sciences, Örebro University, Örebro, Sweden; 2 Department of Laboratory Medicine, Faculty of Medicine and Health, Örebro University, Örebro, Sweden; 3 Department of Women’s Health, Faculty of Medicine and Health, Örebro University, Örebro, Sweden

**Keywords:** Cervical screening, Cytology, DNA methylation, Genotyping, Human papillomavirus

## Abstract

**Background:**

With HPV screening the specificity of screening positives has decreased, even with a cytological triage test. Increases in colposcopies and detection of benign or low-grade dysplasia are reported, not least in older women. These results highlight the necessity to find other triage tests in HPV screening strategies, so that women can be more accurately selected for colposcopy, thus minimizing the clinically irrelevant findings.

**Methods:**

The study included 55- to 59-year-old women who exited the screening with normal cytology, but later in a follow-up test were positive for the HPV genotypes 16, 18, 31, 33, 35, 39, 45, 51, 52, 56, 58, 59, 66 and 68 and had a cervical cone biopsy done. To model a screening situation with hrHPV-positive women, three different triage strategies, namely, cytology, genotyping and methylation, were performed. The study considered the effect of direct referral to colposcopy for HPV genotypes 16, 18, 31, 33, 45, 52 and 58, and methylation for FAM19A4 and hsa-mir124-2 and/or any form of abnormal cytology.

**Results:**

Seven out of 49 women aged 55–59 years with hrHPV had a cone biopsy with high-grade squamous intraepithelial lesion. No triage method found all cases, and when comparing positive and negative predictive value and false negative rate, cytology showed better results than genotyping and methylation.

**Conclusion:**

This study does not support a switch in triage strategies from cytology to hrHPV genotyping and methylation for women above 55 years of age yet, but demonstrates the need for more evidence on molecular triage strategies.

## Introduction

Where cervical screening programmes are implemented cervical cancer is reduced, since cervical cancer is largely preventable through local treatment of screen-detected cervical precursor lesions, cervical intraepithelial neoplasia [1]. Cervical cancer is caused by a persistent infection of certain types of human papilloma virus (HPV) [2]. In several countries a shift is ongoing where HPV testing is replacing cytology in the screening programmes, as this offers greater protection against cervical cancer [3, 4] as well as allowing longer screening intervals [5, 6]. Nevertheless, HPV is a common infection; estimates show that about 10% [7, 8] of the female population at screening age are carriers of the virus, although exact figures differ between continents, age groups and populations.

The HPV test has high sensitivity but lower specificity to detect high-grade squamous intraepithelial lesion (HSIL), compared to cytology. This is due to the fact that HPV infections most often are transient, and only a minor proportion become persistent and cause dysplasia and cancer [4, 9]. Studies show that some HPV genotypes are more common as an infection but not as likely to cause cancer, while other genotypes are more rare but dominate in cervical cancer development [10, 11]. To increase the specificity in the screening, the primary HPV test is followed by a secondary method for triaging. Today, the most commonly used method of triage is cytology. Increases in colposcopies and in detection of benign or low-grade dysplasia with HPV screening compared to cytology screening are reported from the Netherlands among other countries, even though triage is implemented [1, 12, 13]. This lower specificity of the HPV test dictates the necessity of use of other triage tests in current HPV screening strategies, which can select women for colposcopy more accurately [5] and thus minimize the clinically irrelevant findings.

Cytology as a triage method has shown limited value due to its poor sensitivity, not least among the older age cohorts in the screening [14, 15]. The presence of a group of women with HPV-positive, cytology-negative tests in the screening presents a challenge, and so far, the retesting intervals for this group is under careful consideration. Cytology is still not optimal, due to the risk of missing women with HSILs [16]. In addition, many cytological HSILs will regress, as this has been shown to be a heterogeneous group with many cases regressing spontaneously without treatment [17]. Methods for triaging women in the screening programme are in great need, not least among women exiting the screening programme.

Genotyping of HPV could be an alternative triage method in the screening programme, since there is a difference in cancer risk between genotypes, where HPV 16 and 18 genotypes are the most common in cervical cancer [18–20]. A long-term Swedish cohort study shows difference in the oncogenic potential of several HPV types [11], with HPV 16, 18, 31 and 33 having the highest risk of developing histologically confirmed HSIL (CIN 3+), followed by HPV 35, 45, 52 and 58 as intermediate risk and HPV 39, 51, 56, 59, 66 and 68 as lowest risk among high-risk HPV (hrHPV). With minor disparity, this has been confirmed in a meta-analysis from 2020 [21]. Data from Sweden also show that HPV 16, 18, 31, 33, 45 and 52 were found in 83.6% of all cervical cancers in Sweden, and the rest of the genotypes included in screening added only 2.6% of the cancers [10].

Hypermethylation of certain sites in the human genome have been associated with high-grade cervical dysplasia and cervical cancer [22] and could therefore possibly be used for triaging in the screening. Methylation of promoters in the human genes FAM19A4, which perform immunomodulation and influence macrophage activity, and mir124-2, which act as a tumour suppressor, have shown high reproducibility and good correlation to CIN3 and cervical cancer [23, 24]. FAM19A4/miR124-2 methylation analysis has also shown equal sensitivity for CIN3 + and similar negative predictive value (NPV) as cytology [25]. Bimolecular tests to identify hypermethylated FAM19A4 and mir-124-2 have been suggested as a way to increase the specificity in a screening programme with HPV [23, 24].

Studies on screening programmes with HPV as primary test and different molecular strategies are few. Older women in these studies need special attention, since in this age cohort colposcopies are more often inconclusive, cytology is not as reliable and whether reactivation of persistent infections – so-called latent infections – develop in the same pattern as newly acquired infections is not clearly established. Methylation and genotyping are objective methods compared to cytology. They show promising results in increasing the specificity in HPV screening in the same way as cytology, and are appealing, as they can be used on self-samples [25, 26]. The aim of this study was to evaluate triage strategies such as cytology, genotyping and FAM19A4/miR124-2, among HPV-positive women 55–59 years of age.

## Material and method

The study cohort used in this study has previously been published [14]. Briefly, women between 55 and 59 years old who exited the cervical screening programme with normal cytology 2012–2014, in total 2973 women, were invited to participate in that previous study. The biobanked liquid-based cytology samples [27] were analysed for HPV with a DNA-based assay detecting 35 HPV genotypes, both low-risk HPV (lrHPV) and hrHPV, n = 2031.

The current study includes women who participated in the former study and who were positive for the HPV genotypes 16, 18, 31, 33, 35, 39, 45, 51, 52, 56, 58, 59, 66 and 68 in the follow-up test, and had a cervical cone biopsy done at the clinical follow-up. To model a real-life screening situation with hrHPV-positive women, simulation of three different triage strategies – cytology, genotyping and methylation – was performed. The study model considered the effect of direct referral to colposcopy for HPV genotypes 16, 18, 31, 33, 45, 52 and 58 [21], methylation positives and/or any form of abnormal cytology.

This study was approved by the regional ethical committee board in Uppsala, Sweden (D-nr. 2014/121).

ThinPrep cytology slides were assessed by one experienced and certified cytotechnician and classified according to the international Bethesda classification system [28] with atypical squamous cells of undetermined significance (ASC-US); atypical squamous cells, cannot exclude high-grade lesion (ASC-H); low-grade squamous intraepithelial lesion (LSIL); HSIL; squamous cell carcinoma; atypical glandular cells (AGC); adenocarcinoma in situ (AIS); or adenocarcinoma. Concerning histopathology, cone specimens were formalin fixed and paraffin embedded, and thereafter slides were cut at 4 μm and stained with haematoxylin and eosin and evaluated according to present WHO classification [29], by either of two senior pathologists.

DNA was extracted from liquid-based cytology samples using QiaAmp DNA mini kit (Qiagen, Hilden, Germany) [30]. HPV detection and genotyping was performed with CLART (Genomica, Madrid, Spain) according to the manufacturer’s instructions. CLART is a test that detects 35 different HPV genotypes.

For methylation analysis the QIAsure Methylation Test Kit (Qiagen, Hilden, Germany) detecting promoter hypermethylation of the genes FAM19A4 and hsa-mir124-2 was used. Extracted DNA was subjected to bisulphite treatment with EZ DNA Methylation-Goldkit (Zymo Research, Irvine, CA, USA). Detection of hypermethylation in any of the two genes resulted in a positive test result.

### Data and statistical analysis

Excel 2010 (Microsoft, Redmond, WA, USA) was used for data collection and evaluation. The performance was evaluated for three screening strategies: cytology, HPV 16/18/31/33/45/51/58 genotyping and FAM 19A4/has-mir124-2 methylation. Positive predictive value (PPV) and negative predictive value (NPV) were calculated for comparison of the accuracy of the test results between the triage methods. Further analysis of the false negative rate (FNR) was done for comparing the triage methods’ likelihood of improperly indicating no presence of dysplasia. Absolute HSIL risk was calculated for the different screening strategies.

## Results

The study population consisted of 49 hrHPV-positive women from a study of women exiting the screening programme [14].

**Fig. 1:**
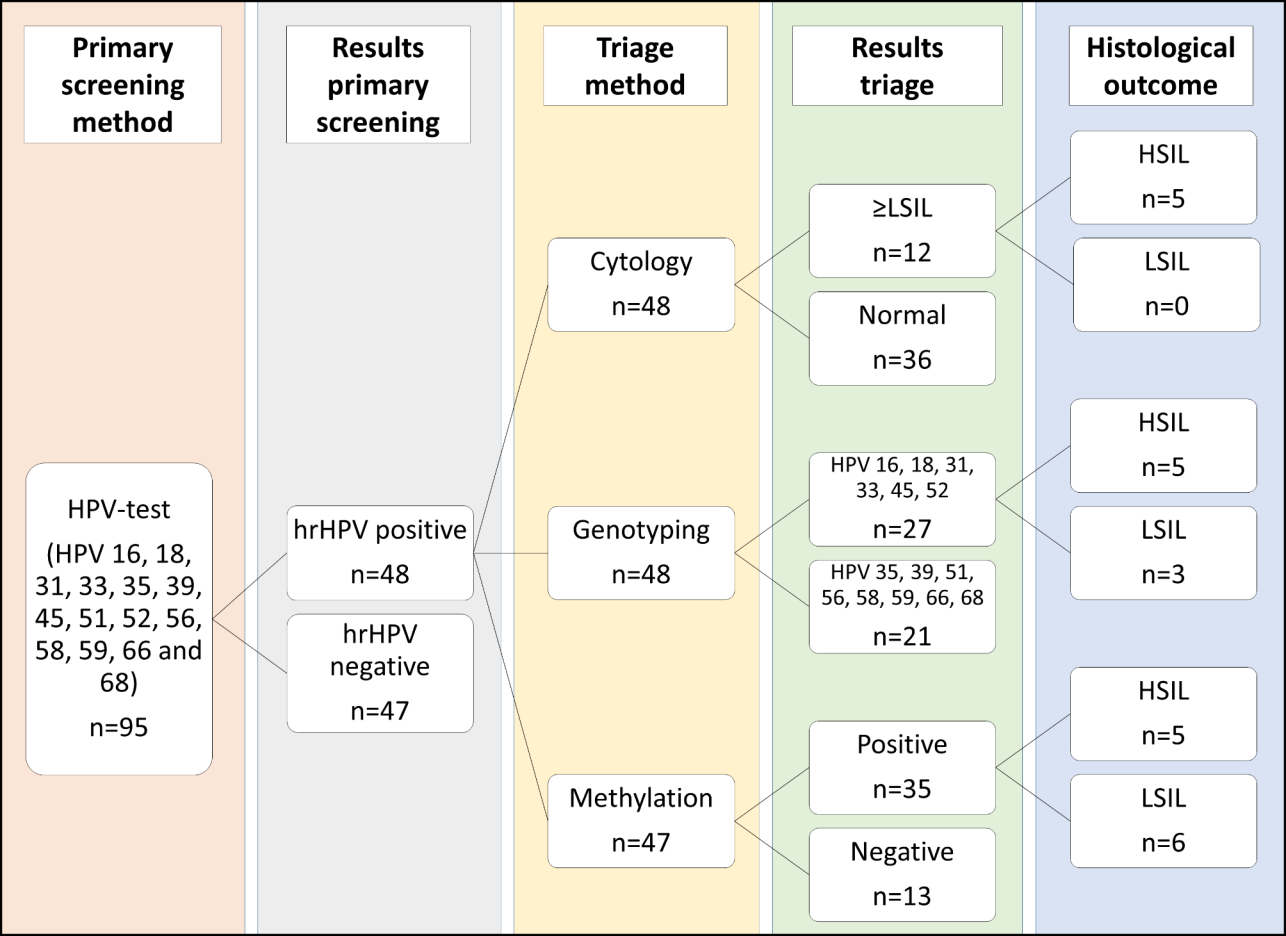
Flowchart of study design and results of the triage methods cytology, genotyping and methylation

Of the women included in this study protocol, 49 were hrHPV-positive for HPV 16, 18, 31, 33, 35, 39, 45, 51, 52, 56, 58, 59, 66 and/or 68 and had a cone biopsy done. The screening samples were genotyped and analysed for cytology and methylation (Fig. 1).

Abnormal cytology was found in 12/49 samples, genotypes resulting in direct colposcopy were detected in 30/49 samples and methylation positivity was found in 35/48 samples; one sample had too little material to analyse after storage in the biobank (Fig. 1; Table 1).

**Table 1:** Results from triaging with cytology, genotyping and methylation

Triage test	hrHPV pos, n	Triage test pos, n	Normal histo, n	LSIL histo, n	NPV ≥LSIL	PPV ≥LSIL	FNR ≥LSIL	HSIL histo, n	NPV ≥HSIL	PPV ≥HSIL	FNR ≥HSIL
Cytology	49	12	7	0	0.73	0.42	67	5	0.95	0.42	29
Genotyping	49	30	20	4	0.74	0.33	33	6	0.95	0.2	14
Methylation	48	35	24	6	0.69	0.31	27	5	0.85	0.14	29

Abbreviations: hrHPV, high-risk HPV; histo, histology; LSIL, low-grade squamous intraepithelial lesion; HSIL, high-grade squamous intraepithelial lesion; NPV, negative predictive value; PPV, positive predictive value; FNR, false negative rate.

A total of seven HSILs were found in this material where no method alone detected all of the cases. Of these, only three were found by all triage methods. Among the 49 women with a positive HPV-test, nine LSILs were detected. Cytology detected none, methylation six and with genotyping including HPV 16, 18, 31, 33, 45, 52 and 58 four cases were found. When including all 14 genotypes, all nine cases were detected and of them two were methylation negative and all had normal cytology.

## Discussion and conclusion

To further develop the HPV-based cervical screening, future steps could include vaginal self-sampling followed by molecular triage using broad genotyping and methylation for risk stratification. In an attempt to address this question in a pilot strategy, we used a cohort of samples including women 55–59 years of age in 2012–2014 who exited the screening programme with a normal cytology and had a positive follow-up sample including HPV genotypes 16, 18, 31, 33, 35, 39, 45, 51, 52, 56, 58, 59, 66 and 68, as well as a clinical follow up with histological cone biopsy [14]. The aim of the study was to compare triage with cytology, HPV genotyping and methylation on the HPV positive samples in women age 55–59 years.

Previous studies show that methylation is non-inferior to cytology triage [25, 31], but some data also indicate that methylation increases spontaneously with age [32, 33]; this must be further investigated before being introduced in the screening for all age groups. For the age group in the current study, as well as in older cohorts, cytology has shown to be inferior in detecting HSIL [14, 16, 34], and in this study, methylation as a triage test is no better than cytology. The NPV as well as the PPV for ≥ HSIL were lower in the methylation triage, 0.85 and 0.14, compared to the cytology triage, 0.95 and 0.42, respectively. This applies both for ≥ LSIL and for ≥ HSIL.

The NPV of hrHPV-positive, methylation-negative samples was 93% for ≥ CIN2 in the article by Bonde et al. [31], whereas in our material the NPV was only 85% for this group. Here, the sample size is, however, very limited, but still the possibility that methylation is not equal in different age groups could be a reason for the results in our study [33]. It is suggested that DNA methylation varies due to a number of factors, including age and disease status. Increased methylation in CpG sites, related to older age, have been shown in different species and is thought to be an example of methylation drift. Other sites may simultaneously be hypomethylated. Findings may also differ between tissue types and contributing factors to methylation changes may as well be found in altered expression of enzymes responsible for adding methylation groups (methyltransferases). The exact biological function of age-increased hypermethylation in genes investigated here such asFAM19A4/miR124-2, involved in immunomodulation and tumour suppressor, is however yet to be determined [35].

Concerning genotyping in screening, a meta-analysis was published in 2020 [19] with results showing high and moderate risk for 16, 18, 31, 33, 45, 52 and 58, which was used in the triage setting in our study. HPV positivity for other than these HPVs showed to be non-inferior to negative cytology in detecting HSIL in this current study population with an NPV of 95%, but with a better FNR than cytology.

This study has not explored a risk stratification with a combination of different triage methods, due to the small study population, but that would be the natural step in continuous work on the subject. With that in mind, a limitation in this study is the few histological HSILs in this study population. This illustrates the need of studies on triage strategies that discriminate different age groups and individualize risk stratification, as well as the need to further investigate the use of combinations of different molecular tests.

## Conclusion

This study does not support a transition to extended genotyping and DNA methylation with these methods at this time. Rather, it supports the need for further studies with larger cohorts, other methylation methods as well as prospective studies that demonstrate real-life outcomes.

## Data Availability

The data contain potentially identifying and sensitive patient information according to the EU data protection act (GDPR) and the Swedish act concerning health care data (SFS 2008:355). Thus, availability for research purposes requires ethical vetting, which must be initiated by the organization responsible for the data. Contact can be initiated by contacting the researcher or the organization, dso@regionorebrolan.se (data safety officer).

## List of Abbreviations

AGC: Atypical glandular cells
AIS: Adenocarcinoma in situ
ASC-H: Atypical squamous cells cannot exclude high-grade lesion
ASC-US: Atypical squamous cells of undetermined significance
FNR: False negative rate
HPV: Human papilloma virus
hrHPV: High-risk human papilloma virus
HSIL: High-grade squamous intraepithelial lesion
lrHPV: Low-risk human papilloma virus
LSIL: Low-grade squamous intraepithelial lesion
NPV: Negative predictive value
PPV: Positive predictive value
